# Centromere sliding on a mammalian chromosome

**DOI:** 10.1007/s00412-014-0493-6

**Published:** 2014-11-21

**Authors:** Stefania Purgato, Elisa Belloni, Francesca M. Piras, Monica Zoli, Claudia Badiale, Federico Cerutti, Alice Mazzagatti, Giovanni Perini, Giuliano Della Valle, Solomon G. Nergadze, Kevin F. Sullivan, Elena Raimondi, Mariano Rocchi, Elena Giulotto

**Affiliations:** 1 Dipartimento di Farmacia e Biotecnologie (FABIT), Università di Bologna, Bologna, Italy; 2 Dipartimento di Biologia e Biotecnologie “Lazzaro Spallanzani”, Università di Pavia, Pavia, Italy; 3 Centre for Chromosome Biology, School of Natural Sciences, National University of Ireland, Galway, Ireland; 4 Dipartimento di Biologia, Università di Bari, Bari, Italy

## Abstract

The centromere directs the segregation of chromosomes during mitosis and meiosis. It is a distinct genetic locus whose identity is established through epigenetic mechanisms that depend on the deposition of centromere-specific centromere protein A (CENP-A) nucleosomes. This important chromatin domain has so far escaped comprehensive molecular analysis due to its typical association with highly repetitive satellite DNA. In previous work, we discovered that the centromere of horse chromosome 11 is completely devoid of satellite DNA; this peculiar feature makes it a unique model to dissect the molecular architecture of mammalian centromeres. Here, we exploited this native satellite-free centromere to determine the precise localization of its functional domains in five individuals: We hybridized DNA purified from chromatin immunoprecipitated with an anti CENP-A antibody to a high resolution array (ChIP-on-chip) of the region containing the primary constriction of horse chromosome 11. Strikingly, each individual exhibited a different arrangement of CENP-A binding domains. We then analysed the organization of each domain using a single nucleotide polymorphism (SNP)-based approach and single molecule analysis on chromatin fibres. Examination of the ten instances of chromosome 11 in the five individuals revealed seven distinct ‘positional alleles’, each one extending for about 80–160 kb, were found across a region of about 500 kb. Our results demonstrate that CENP-A binding domains are autonomous relative to the underlying DNA sequence and are characterized by positional instability causing the sliding of centromere position. We propose that this dynamic behaviour may be common in mammalian centromeres and may determine the establishment of epigenetic alleles.

**Electronic supplementary material:**

The online version of this article (doi:10.1007/s00412-014-0493-6) contains supplementary material, which is available to authorized users.

## Introduction

Centromeres are genetic loci whose identity depends not on the sequence of DNA on which they are formed but on a specific nucleosome configuration containing the centromere-specific histone H3, centromere protein A (CENP-A) (Sullivan 2001; Black and Cleveland 2011). Centromere-associated DNA varies widely in different species and even within a karyotype, but the core protein composition, based on the presence of CENP-A nucleosomes, is a universal feature of eukaryotic chromosomes (Malik and Henikoff 2009). Both CENP-A and its deposition machinery, comprising a distinct pathway for chromatin assembly, are highly conserved during evolution (Maddox et al. 2012; Kato et al. 2013). Precisely how this chromatin architecture is related to its underlying DNA is still poorly understood. Typically, mammalian centromeres are associated with highly repetitive tandem satellite arrays which have limited the detailed molecular dissection of this critical chromatin domain (Karpen and Allshire 1997). Taking advantage of the presence of two alpha satellite subfamilies at the centromere of human chromosome 17, Maloney and colleagues (Maloney et al. 2012) showed that the centromeric function can be linked to different repeated sequence variants generating ‘functional epialleles’.

Separation of centromere identity from DNA sequence was first inferred from the analysis of human neocentromeres, in which centromeres form on single-copy sequences in rearranged chromosomes (Barry et al. 1999). Human neocentromeres have been identified in clinical cytogenetic laboratories; most of them arose to stabilize otherwise acentric fragments while a less common type was found in intact chromosomes where the native centromere has been inactivated giving rise to neodicentrics (Marshall et al. 2008). Given the lack of satellite repeats, some human neocentromeres have been deeply analysed by chromatin immunoprecipitation approaches (ChIP-on-chip or ChIP-seq) (Chueh et al. 2005; Alonso et al. 2010; Hasson et al. 2011, 2013); the main conclusions of these studies were that CENP-A binding is largely independent of DNA sequence and that extended herochromatin domains are not required for centromere function. Neocentromere formation on rearranged or engineered chromosomes has also been observed in other species, including *Saccharomyces pombe* (Steiner and Clarke 1994), *Drosophila melanogaster* (Williams et al. 1998), *Candida albicans* (Ketel et al. 2009), maize (Fu et al. 2013) and chicken (Shang et al. 2013).

The formation of novel centromeres can also occur during evolution through the repositioning of the centromere to a new site without chromosomal rearrangement; these evolutionary new centromeres (ENCs) significantly impact karyotype evolution, but their mechanisms of formation are unknown (Kalitsis and Choo 2012; Rocchi et al. 2012). Originally described in primates (Montefalcone et al. 1999), ENCs are particularly prevalent in the genus *Equus* (horses, asses and zebras) (Carbone et al. 2006). Although the majority of ENCs so far described contains satellite DNA arrays, it was proposed that the initial seeding of a new centromere during evolution occurs within an anonymous genomic region and that the acquisition of tandem repeats is a late phenomenon (Amor and Choo 2002; Piras et al. 2010); recent data on rice centromeres suggest that satellite repeats may evolve to stabilize centromeric nucleosomes (Zhang et al. 2013). The rapidly evolving *Equus* species gave us the opportunity to catch snapshots of evolutionarily new centromeres in different stages of ‘maturity’ (Piras et al. 2010). A multistep model for the birth, evolution and complete maturation of ENCs was proposed: The first step would consist in the shift of the centromeric function to a new position lacking satellite DNA, while the satellite DNA from the old centromere remains in the ancestral position; a subsequent step would be the loss of the leftover satellite DNA; finally, at a later stage, satellite repeats would colonize the new centromere giving rise to completely ‘mature’ centromeres (Amor and Choo 2002; Piras et al. 2010). During this process, dicentric chromosomes may be transiently generated but, according to the model, epigenetic marks rather than specific DNA sequences may determine the switch of the centromeric function from the old to the new position. Alternatively, the old centromere may be physically lost through chromosome rearrangement, similarly to what has been observed in clinical neocentromeres. A clear example of evolutionarily young neocentromere is the one on horse chromosome 11 which is completely devoid of satellite DNA (Wade et al. 2009). A ChIP-on-chip analysis of this centromere in one individual revealed the presence of two CENP-A binding domains. In order to shed light on the organization of the centromeric function in horse chromosome 11, in the present work, we exploited this satellite-less centromere to examine the detailed functional organization of this native mammalian centromere by analysing five new individuals. We demonstrated that the centromeric function is not fixed and identified at least seven functional alleles scattered in a region of about 500 kb; this surprisingly high positional variation gives rise to multiallelic epigenetic polymorphism. At a molecular level, these results reveal a mobility of CENP-A nucleosome arrays, a property that could be related to the evolutionary mobility of centromeres.

## Materials and methods

### Horse cells

Primary fibroblast cell lines were obtained from the skin of five different slaughtered animals and designated for convenience HSF-B, HSF-C, HSF-D, HSF-E and HSF-G. We do not know to which breed these animals belong. We tested their relatedness by standard DNA typing using the following microsatellite loci: AHT4, AHT5, ASB2, ASB17, ASB23, CA425, HTG4, HTG6, HTG7, HTG10, HMS2, HMS3, HMS6, HMS7, VHL20, HMS1. These include nine loci recommended by the ‘Equine Genetics and Thoroughbred Parentage Testing Standardization Committee’ of the International Society for Animal Genetics (ISAG) and eight additional loci commonly used for horse parentage testing and identification (Equine Gentypes Panel 1.1, Thermo Scientific). We then tested likelihood of relation using the Familias 3.1.3 software (http://familias.no).

The cells were cultured in high glucose DMEM (EuroClone) medium supplemented with 15 % foetal bovine serum, 2 mM L-glutamine, 1 % penicillin/streptomycin and 2 % non-essential amino acids at 37 °C with 5 % CO_2_. The cell lines were from three male (HSF-B, HSF-C and HSF-G) and two female (HSF-D and HSF-E) animals. Cytogenetic analysis demonstrated that all cell lines had a diploid modal chromosome number (64) and a normal karyotype (Supplementary Fig. 1).

### ChIP and ChIP-on-chip analysis

To identify the sequences bound by CENP-A, native chromatin immunoprecipitation analysis was performed, as previously described (Wade et al. 2009). Briefly, native chromatin was prepared from horse fibroblasts by nuclease digestion of cell nuclei; immunoprecipitation was then performed using a polyclonal antibody against the centromeric protein CENP-A (Trazzi et al. 2009). We have previously demonstrated that this antibody is able to recognize horse centromeres (Wade et al. 2009). Both input and immunoprecipitated DNA fragments were purified and amplified using the whole genome amplification (WGA) kit (Sigma-Aldrich, St. Louis, USA). ChIPed DNA was analysed by real-time PCR before and after WGA amplification.

The input and the immunoprecipitated DNAs were co-hybridized to a NimbleGen custom tiling array containing a 3.2 Mb region between nucleotides ECA11:25,566,599-28,305,611 with an average resolution of 100 bp. The array data were deposited in NCBI’s Gene Expression Omnibus, and they are accessible through GEO Series accession number GSE57986 (http://www.ncbi.nlm.nih.gov/geo/query/acc.cgi?acc=GSE57986). DNA binding peaks were identified by using the statistical model and methodology described at (http://chipanalysis.genomecenter.ucdavis.edu/cgi-bin/tamalpais.cgi) (Bieda et al. 2006) using stringent parameters for peak identification (98th percentile threshold and *p* < 0.0001).

### Real-time PCR analysis

Real-time PCR was performed using the Go Taq qPCR Master Mix (Promega) on a DNA Engine Opticon 2 System (Bio-Rad). Data were analysed using the Opticon Monitor 3 software.

For each individual, two independent real-time PCR experiments were performed on immunoprecipitated and input DNA using the primer pairs spanning the region of interest listed in Supplementary Table 1. The single-copy gene PRKCi (gene ID: 100063737, forward primer: TGGAGCAAAAGCAGGTGGTA, reverse primer: ATCGTCATCTGGAGTGAGCTG) was used as control. Real-time PCR was performed using the following temperature program: initial denaturation at 95 °C for 2 min; 50 cycles with denaturation at 95 °C for 15 s, annealing at 61 °C for 30 s and elongation at 72 °C for 30 s. Fluorescence detection was performed for 15 s at 80 °C. Final extension at 72 °C for 5 min. For melting curve analysis, a temperature gradient (60–94 °C, 1 °C/s) was applied. Each reaction was carried out in triplicate. For each primer pair, relative standard dilutions of input DNA (1:1, 1:10, 1:100) were included in the experiments. Real-time PCR results were considered reliable only when the *r* ^2^ value of the calibration curve was comprised between 0.95 and 1. To evaluate the relative fold enrichment, the ΔΔCt formula was applied where Ct is the cycle threshold.

### SNP analysis

SNPs used for the analysis were identified using the website (http://www.broadinstitute.org/mammals/horse/snp).

Firstly, the SNPs were tested on genomic DNA by PCR and sequencing. Genomic DNA was extracted from primary fibroblasts using QIAGEN Blood and Cell culture DNA Midi kit according to manufacturer’s instructions. DNA was amplified using the High Fidelity Herculase II Fusion DNA Polymerase (Stratagene, Agilent Technologies), and PCR products were sequenced. SNPs that were heterozygous in genomic DNA (Supplementary Table 2) were analysed both on input and on immunoprecipitated DNA from ChIP experiments.

### BAC clones

The DNA segment spanning the centromere of horse chromosome 11 (chr11:27,400,000–28,150,000) was derived from the EquCab2.0 horse genome sequence assembly. The sequence was used as query against NCBI *Equus caballus* Clone End Sequence database. Bacterial artificial chromosome (BAC) end sequences from the horse CHORI-241 BAC library were searched (Leeb et al. 2006). The seven selected clones are reported in Supplementary Fig. 2. Their cytogenetic position was validated by fluorescent in situ hybridization (FISH) on horse metaphase chromosomes (Supplementary Fig. 3).

### Immuno-FISH on extended chromatin fibres

Extended chromatin fibres were prepared using published methods (Lam et al. 2006; Maloney et al. 2012) with slight modifications; in particular, an electrical device, equipped with a pulley, was built specifically to raise slides from the lysis buffer perpendicularly and at a constant speed. Immunofluorescence, carried out using a CREST serum (kindly provided by Claudia Alpini, Fondazione I.R.C.C.S. Policlinico San Matteo, Pavia), was followed by FISH with the appropriate BAC clones. Fibres were prepared from at least two independent experiments; combined immunostaining and FISH were performed using different schemes to avoid potential hybridization or detection bias with fluorescent secondary antibodies. DNA fibres were counterstained with 5 mg/mL DAPI and mounted with DAKO mounting medium (DAKO).

### Animal rights statement

The horse skin samples were taken from animals not specifically sacrificed for this study; the animals were being processed as part of the normal work of the abattoirs.

## Results

### Variable position of CENP-A binding domains in different individuals

**Fig. 1:**
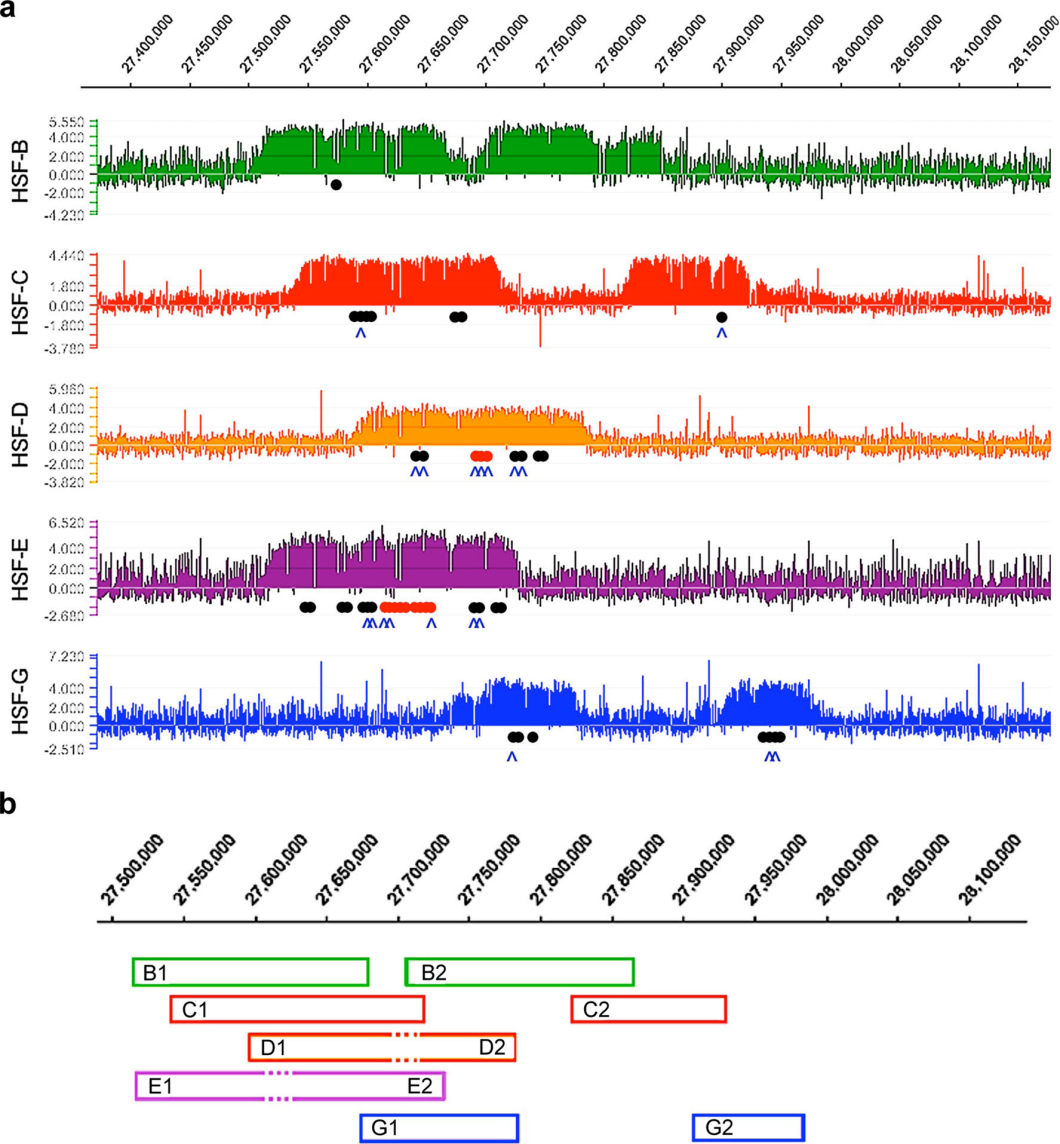
Variable position of the centromere of horse chromosome 11. **a** DNA obtained by chromatin immunoprecipitation. Using an anti-CENP-A antibody, from five different horse fibroblast cultures was hybridized to a tiling array covering the centromere region. Results are presented as the log2 ratio of the hybridization signals obtained with immunoprecipitated DNA versus input DNA; *x*-axis, genomic coordinates on ECA11. Positions of informative SNPs are indicated as *black dots* (a single nucleotide of the SNP is enriched in immunoprecipitated DNA), *red dots* (both SNP alleles are present in immunoprecipitated DNA) and *blue carats* (SNPs shown in Fig. 3). **b** Peak positions are represented as *boxes*. Epiallele identification was obtained by combining ChIP-on-chip, SNP (Fig. 3) and fibre FISH (Fig. 4 and Supplementary Table 2) results. Sequence coordinates refer to the horse EquCab2.0 (2007) sequence assembly, as reported by the UCSC genome browser (http://genome.ucsc.edu). Alleles are designated by the letter of the horse they derive from, followed by ‘1’ or ‘2’ to distinguish the two variants. In HSF-D and HSF-E, where a single broad peak was identified by ChIP-on-chip while two distinct centromeric domains were identified by fibre-FISH (Fig. 4) and SNP analysis (Fig. 3 and Supplementary Table 2), *dotted lines* represent the region of overlap of the two binding domains in the reference sequence. Therefore, at least seven different centromeric domains can be identified: Ba/Ea, Bb, Ca, Cb, Da/Eb, Db/Ga, Gb

We established fibroblast cell lines from five horses (HSF-B, HSF-C, HSF-D, HSF-E and HSF-G). Using 17 microsatellite loci (Thermo Scientific Equine Genotypes Panel 1.1), we determined their likelihood of relation with the Familias 3.1.3 software, demonstrating that they were unrelated (see Materials and Methods). The unexpected observation of two CENP-A binding domains in the horse previously analysed (Wade et al. 2009) prompted us to extend the analysis to these five new individuals. Chromatin was immunoprecipitated with an antibody against CENP-A. DNA was then purified and hybridized to a 3.2 Mb tiling array (accession number: http://www.ncbi.nlm.nih.gov/geo/query/acc.cgi?acc=GSE57986) spanning the centromeric region of horse chromosome 11 that we previously defined (Wade et al. 2009). The absence of satellite repeats at this locus (Wade et al. 2009) allowed us to position CENP-A-associated DNA (Fig. 1a). Strikingly, each individual exhibited a distinct arrangement of CENP-A binding domains. These were located across a region of approximately 500 kb, with some individuals (HSF-B, HSF-C and HSF-G) exhibiting two clearly defined peaks while others (HSF-D and HSF-E) showed one.

**Fig. 2:**
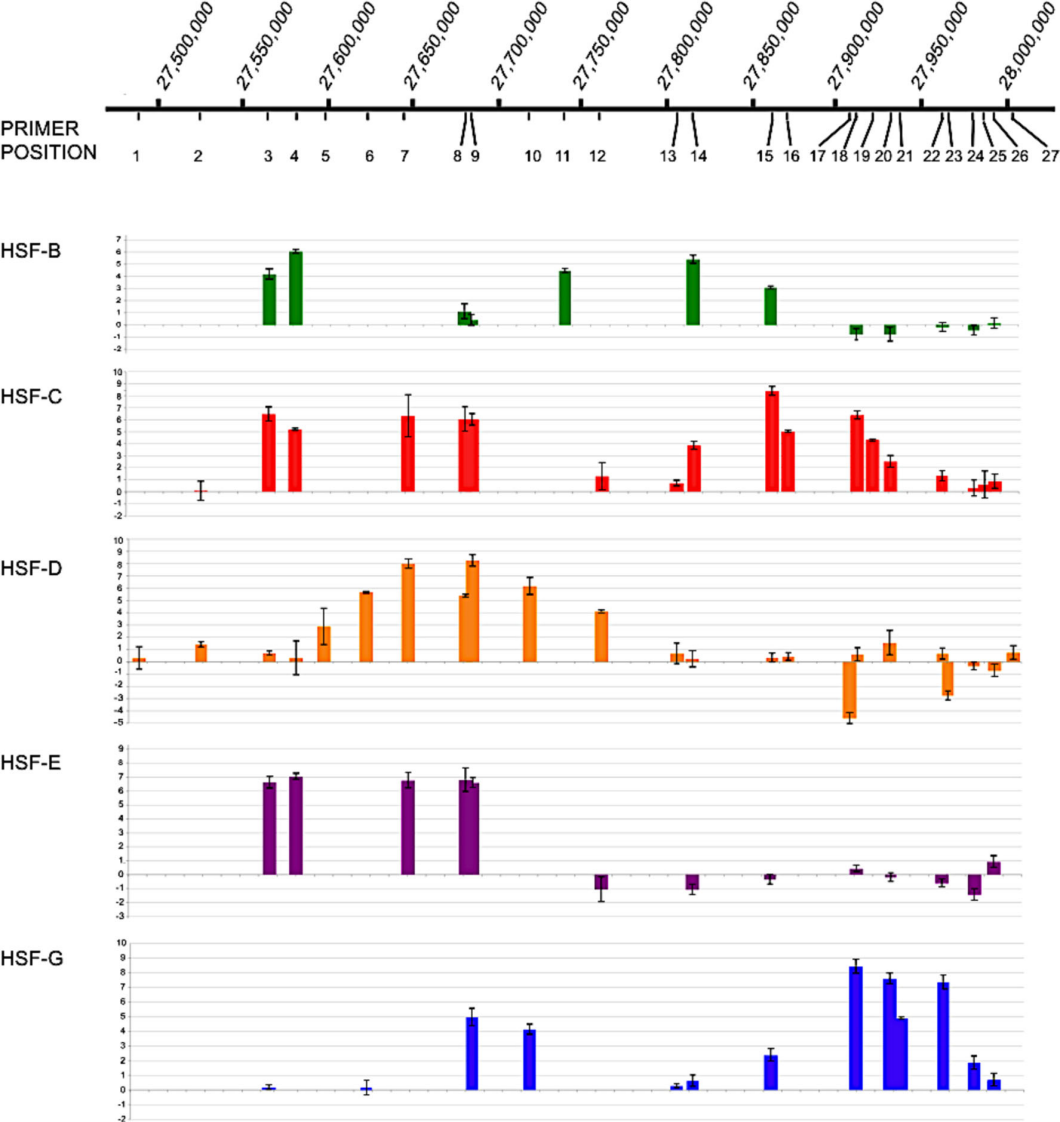
Real-time PCR analysis of the ChIP-on-chip samples. For each cell line (HSF-B, HSF-C, HSF-D, HSF-E and HSF-G), results are presented as the logarithm of the difference between the cycle threshold obtained with the CENP-A immunoprecipitated sample and the cycle threshold obtained with input sample, normalized for the control region (chr11:28,227,839-28,227,938). The *x*-axis shows the genomic position of each primer pair along chromosome 11

**Fig. 3:**
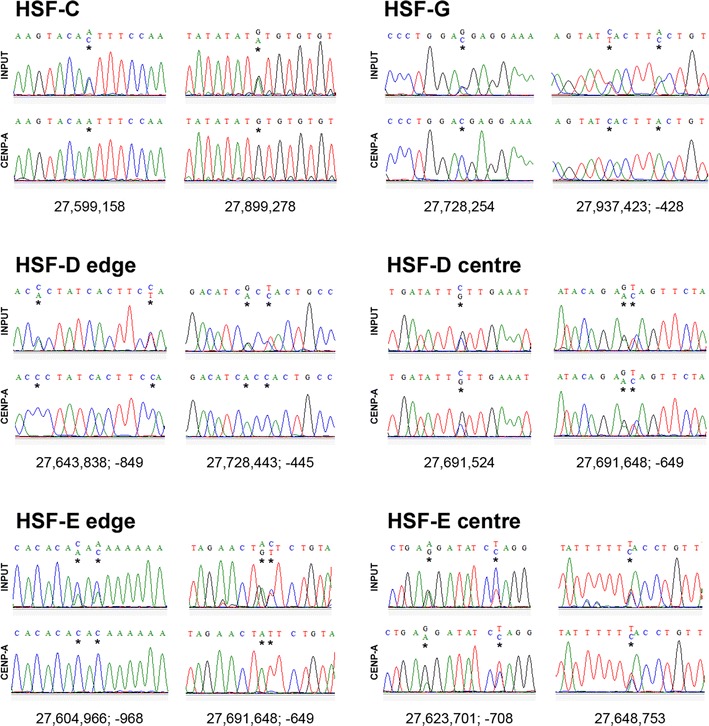
SNP analysis of centromeric domains. Sanger sequence traces from input (*above*) and CENP-A immunoprecipitated (*below*) samples from HSF-C, HSF-G, HSF-D and HSF-E. SNP coordinates are beneath traces. Stars indicate SNPs. For HSF-C, HSF-G, HSF-D-edge and HSF-E-edge, both nucleotides are present in input DNA while the immunoprecipitated DNA is enriched for one of the two nucleotides. For HSF-D centre and HSF-E centre, the two nucleotides are present in both input and CENP-A immunoprecipitated samples

**Fig. 4:**
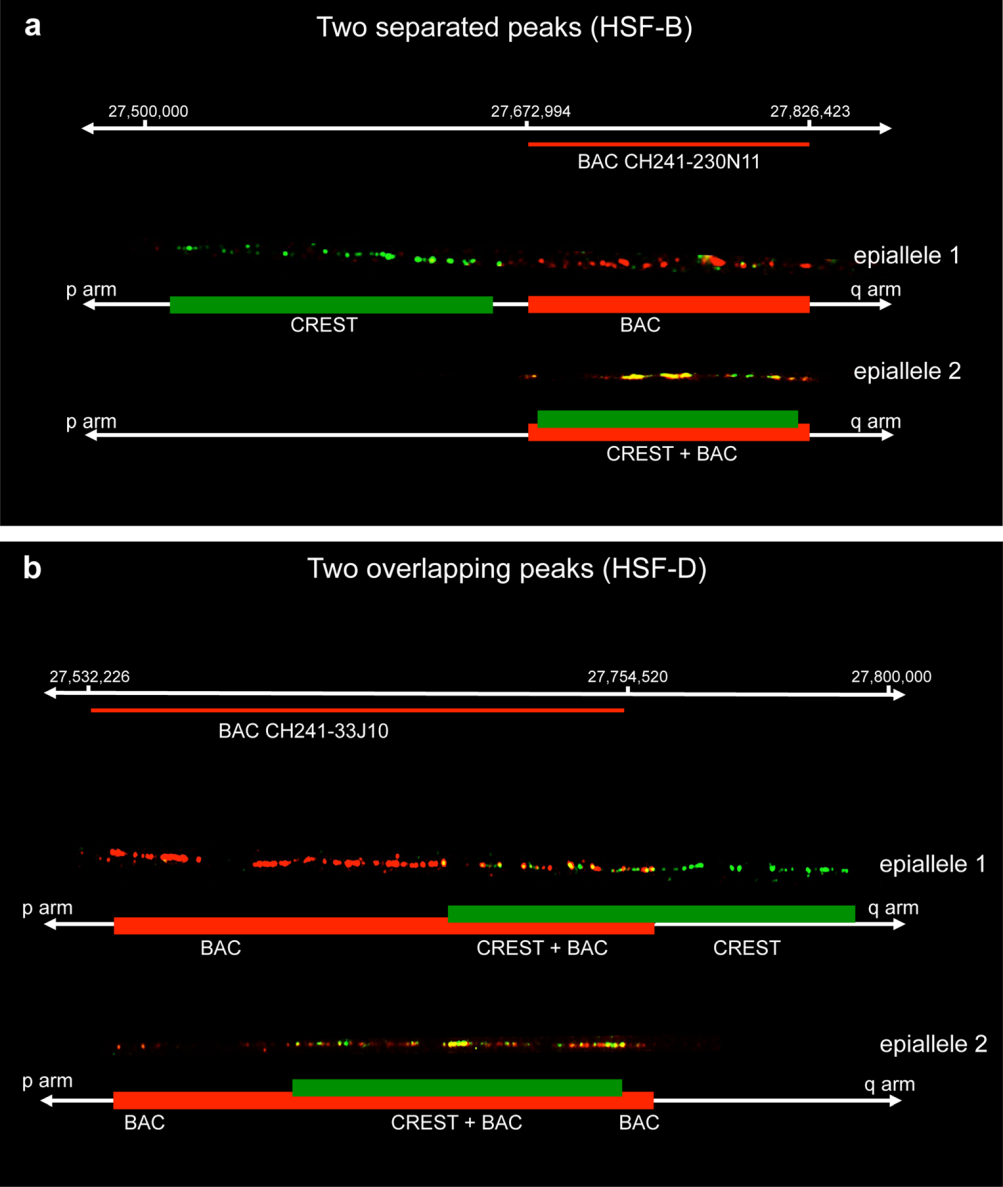
Single molecule analysis of centromeric epialleles on chromatin fibres by immuno-FISH. **a** Organization pattern of functional allelels in horses displaying two separated ChIP-on-chip peaks (HSF-B and HSF-G). The example shown refers to horse HSF-B, and the BAC used was CH241-230 N11. **b** Pattern of functional alleles organization in horses displaying two overlapping ChIP-on-chip peaks (HSF-D and HSF-E). The example shown refers to horse HSF-D, and the BAC used was CH241-33 J10. At the *top* of each panel, the coordinates of the regions occupied by the centromeric domains are reported, and BAC coverage is represented by a *red line*. CREST immuno-staining is green labelled while the BAC FISH signals are *red* labelled. Under each fibre image, a schematic representation is depicted with *green rectangles* corresponding to centromeric domains and *red rectangles* indicating BAC hybridization. Two (HSF-B and HSF-G) or three (HSF-D and HSF-E) independent experiments were performed for each horse, and at least 10 chromatin fibres were analysed. The ratio of epialleles 1 and 2 observed in the individual horses was close to 50 %: HSF-B 5/11 vs 6/11; HSF-G 4/10 vs 6/10; HSF-D 14/26 vs 12/26; HSF-E 6/16 vs 10/16

At least seven functional epialleles were identified in the five horses and are sketched in panel b of Fig. 1; identification was obtained by combining the results of ChIP-on-chip (panel a), qPCR (Fig. 2), SNP analysis (Fig. 3) and fibre immuno-FISH (Fig. 4). Each epiallele occupies about 80–160 kb. These results demonstrate that the centromeric domain of horse chromosome 11 is characterized by great positional variation giving rise to ‘epigenetic polymorphism’. No functionally homozygous individuals were observed; therefore, in spite of our limited sample size, we can infer that this epigenetic locus is highly polymorphic.

To define the position and number of CENP-A binding domains with a different approach, we designed a set of 27 primer pairs (Supplementary Table 1) spanning the 500 kb region. Real-time PCR experiments were then carried out on the DNA purified from CENP-A immunoprecipitated chromatin from the five individuals. The q-PCR data confirmed those obtained by the ChIP-on-chip (Fig. 2): Two regions of CENP-A binding were identified in individuals HSF-B, HSF-C and HSF-G, while a single region could be observed in HSF-D and HSF-E.

### Analysis of domain organization by single nucleotide polymorphism and immuno-FISH on chromatin fibres

The presence of two domains of CENP-A binding in some individuals could reflect a multidomain centromere structure, shared by both chromosomes 11; alternatively, one of the domains seen in HSF-B, HSF-C and HSF-G could be located on one of the two homologous chromosomes 11 and the second one on the other homolog. To unravel which one of the two possibilities was correct, we sought heterozygous nucleotide positions, SNPs, located within the centromeric domains using the SNP database (see Materials and Methods). Informative SNPs were then identified within the CENP-A binding domains of individuals HSF-D, HSF-G and HSF-E. For HSF-C and HSF-B, the SNPs available in the database were not informative; therefore, in these two horses, informative loci were identified by sequencing PCR products from genomic DNA. These heterozygous positions (Supplementary Table 2 and Fig. 1 black and red dots) would allow us to resolve the two homologs in DNA purified from CENP-A chromatin immunoprecipitations: If the two CENP-A domains were present on both homologs, the immunoprecipitated chromatin would contain similar amounts of the two alleles; on the contrary, if each homolog contained a single CENP-A domain, only one of the two alleles would be enriched in the immunoprecipitated chromatin. The results of all experiments relative to the five horses are summarized in the Supplementary Table 2. In Fig. 3, representative Sanger sequence traces from horses HSF-C, HSF-D, HSF-E and HSF-G are shown.

In Fig. 3 (top panels), Sanger sequence traces from input and CENP-A immunoprecipitated DNA, relative to three SNPs in HSF-G and two SNPs in HSF-C are shown. The position of these SNPs is marked with blue carats in Fig. 1 and are listed, using blue colour, in Supplementary Table 2. At all these SNP positions, both nucleotides were present in input DNA while in the immunoprecipitated DNA, enrichment of only one nucleotide was clearly detected. These results strongly suggest that, in HSF-C and HSF-G, each homolog contains a single CENP-A binding domain. Similarly, in HSF-B, the analysis of the heterozygous microsatellite locus strongly suggests that each one of the two CENP-A domains is located on one homolog (Supplementary Table 2).

In HSF-D and HSF-E, in which a single broad peak of CENP-A binding was observed by ChIP-on-chip (Fig. 1) and q-PCR (Fig. 2), different results were obtained when SNPs at the edges (black dots in Fig. 1) or at the centre (red dots in Fig. 1) of the peak were analysed (Fig. 3). At the edges, in DNA purified from CENP-A immunoprecipitations, a single nucleotide was enriched in the sequence profiles, similarly to what we observed within the HSF-C and HSF-G peaks; on the contrary, at the centre of the broad peak, both SNP nucleotides were bound by CENP-A. The interpretation of this result is that CENP-A binds to different regions in the two homologs, as in horses HSF-C and HSF-G. However, in HSF-D and HSF-E, the CENP-A binding domains are partially overlapping in the horse genome sequence and correspond to the left and the right part of the broad ChIp-on-chip peak, respectively; the overlapping region roughly corresponds to the centre of the broad peak. Therefore, also for HSF-D and HSF-E, the results are consistent with the presence of one CENP-A binding domain on each homolog.

The results of SNP analysis were confirmed by an independent approach that is single molecule analysis of centromeric domains by immuno-FISH on chromatin fibres. BACs covering the centromeric domain (Supplementary Fig. 2), as determined by ChIP-on-chip, were used as FISH probes, and a CREST serum was used to detect the functional centromeric domain. In Supplementary Fig. 2a, the BAC clones are listed with their genomic coordinates and their position on the genome map is sketched in panel b of the same figure. Concerning the CREST serum used, we showed that the signals obtained on DNA fibres is perfectly overlapping with the signal obtained by a monoclonal anti-CENP-A antibody (Supplementary Fig. 4), the CREST serum signal being particularly intense and therefore more suitable for the immuno-FISH experiments in combination with BAC clones. Samples from HSF-B, HSF-D, HSF-E and HSF-G were analysed. We observed two different organization patterns of FISH and immuno-staining fluorescent signals which are exemplified in Fig. 4. The first type of arrangement is reported in Fig. 4a and was observed in samples from horses displaying two clearly separated ChIP-on-chip peaks (HSF-B and HSF-G). Two distinct epialleles could be distinguished, one of which (epiallele 1 in Fig. 4a) had the immuno-staining flanking the FISH signal while in the other one (epiallele 2 in Fig. 4a), the immuno-staining and FISH signals were superimposed. The second type of arrangement, observed in horses HSF-D and HSF-E, is reported in Fig. 4b. These two horses displayed a single broad ChIP-on-chip peak, and SNP data indicated that the broad peak was the result of the partial overlap of two distinct peaks. Immuno-FISH confirmed this interpretation: Indeed, as shown in Fig. 4b, two functional alleles could be observed also in these horses. In one epiallele (epiallele 1 in Fig. 4b), the immuno-staining partially covered the FISH signal and extended in the flanking region, while in the other epiallele (epiallele 2 in Fig. 4b), the immuno-staining covered the FISH signal. The immuno-labelled regions of epiallele 1 and epiallele 2 were partially overlapping.

### Sequence analysis of the DNA region containing the CENP-A binding domains

**Fig. 5:**
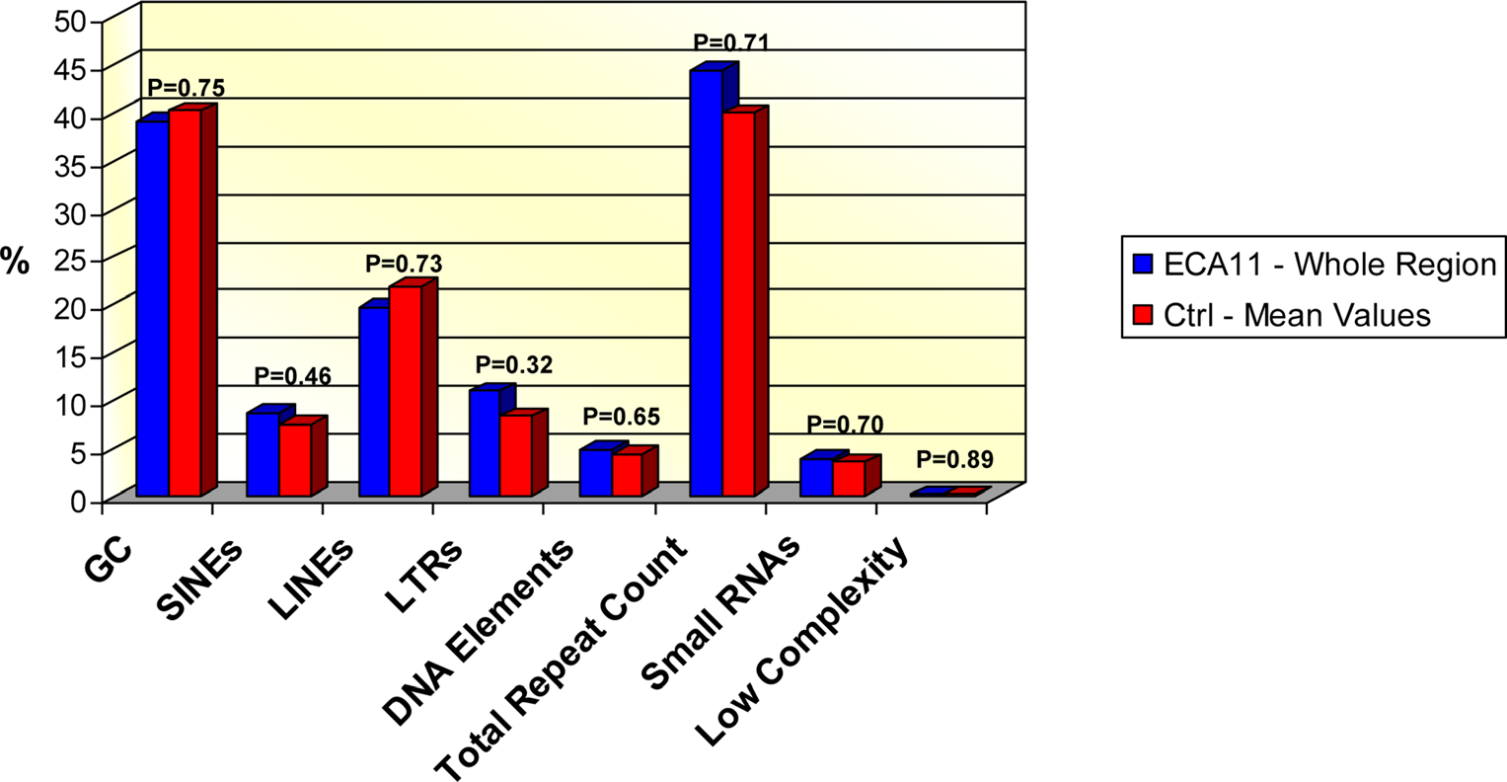
Sequence analysis. *Blue bars* represent the percentage of each class of sequence in the entire ECA11 centromeric region. *Red bars* correspond to mean values from 64 control regions of the percentage of each sequence class (data are reported in Supplementary Table 3). Satistical analysis was performed using the Student’s *t* test, and the *p* values are reported for each comparison, indicating that the differences between the centromeric and the control regions are not statistically significant

To test whether any peculiar DNA sequence composition may account for the presence of centromeric domains, we carried out a detailed analysis of the region under study and of 64 control regions (two interstitial regions from each horse chromosome were chosen at random) of the same size, using the RepeatMasker software (http://www.repeatmasker.org/cgi-bin/WEBRepeatMasker); subtelomeric and heterochromatic regions were intentionally excluded from the analysis. The results are reported in Supplementary Table 3 and summarized in Fig. 5. For ECA11, the analysis was performed on the entire centromeric region and on each individual CENP-A binding domain identified by ChIP-on-chip. In the control regions, the guanine-cytosine (GC) content ranged between 34.74 and 48.52 % with a mean value of 40.25 %. Consistently, in the entire centromeric region, the GC content was 39.12 %; little variation around this value was observed among single CENP-A binding domains (data on peaks in Supplementary Table 3). It is important to note that the GC content of the entire region does not correspond to the mean of the single peaks due to peak overlapping. Student’s *t* test indicated that the average GC content of the control regions was not significantly different from the ECA11 centromeric region (*p* = 0.75, Fig. 5). A similar comparison was carried out for the following classes of repetitive elements: SINEs, LINEs, LTRs, DNA transposable elements, small RNAs and low-complexity repeats; *p* values comprised between 0.32 and 0.89 indicated that the repeated element composition of the ECA11 centromeric domain was comparable to that of the control regions.

## Discussion

The results presented here reveal a remarkable plasticity of the satellite-less centromere of horse chromosome 11. In this analysis of ten horse chromosomes 11, at least seven distinct CENP-A binding domains, each one extending for about 80–160 kb, were found across a region of about 500 kb. These results demonstrate that, in a native mammalian centromere, the positioning of CENP-A binding domains is unrelated to the sequence of the DNA the centromere is associated with and that centromere position can be flexible across a relatively wide single-copy genomic region. Indeed, the sequence features (GC and repetitive elements content) of the ECA11 centromeric region are comparable to those of random intra-chromosomal genomic regions. The analysis of the GC content of this genomic region was performed taking into consideration the isochore theory (Bernardi 1993). According to this theory, stretches of more than 300 kb, uniform for GC content, characterize the genomes of ‘worm-blooded’ vertebrates. With this analysis, we intended to test whether the centromeric region of horse chromosome 11 was inserted in an AT reach isochore, as previously suggested for other mammalian neocentromeres (Marshall et al. 2008).

Although the size and organization of mammalian and fission yeast centromeres are remarkably different, it was recently shown that, also in the small centromere of *S. pombe*, the positioning of CENP-A/Cnp1 nucleosomes varies relative to the underlying DNA sequence among genetically homogeneous cell lines (Yao et al. 2013).

When neocentromeres were experimentally induced in chiken DT40 cells, most of them were formed at multiple positions close to the original centromere; interestingly, detectable levels of CENP-A were found in a 2 Mb region surrounding the original centromere (Shang et al. 2013). The proposed hypothesis was that epigenetic marks favouring ‘centromerization’ were present around the original centromere, and this may be the reason why neocentromeres were preferentially seeded in that region. In spite of the positional variation of neocentromeres induced by chromosome engineering, in the chicken system, centromere spreading seems to be prevented in wild-type cells (Shang et al. 2013). Here, we demonstrated that the wild-type centromere of horse chromosome 11, unlike chicken wild-type centromeres, moves considerably within a 500 kb region. It is important to underline that we analysed an evolutionary neocentromere that was established about one million years ago, after the divergence of horses from the other species of the genus *Equus* (asses and zebras) (Piras et al. 2010). The centromeric domains detectable nowadays are the result of a positional sliding that occurred during the evolution of the horse lineage; we are therefore taking a ‘snapshot’ of an ongoing evolutionary process whose initial shots are unavailable.

It is possible that removal of the centromere of horse chromosome 11 from a typical heterochromatic environment has revealed or exacerbated an underlying dynamic behaviour of CENP-A chromatin, as proposed for experimentally induced neocentromeres in *Drosophila* (Maggert and Karpen 2001). Some human neocentromeres have been shown to be very poor in heterochromatin, and this feature has been correlated with defects of sister chromatid cohesion (Alonso et al. 2010). This observation is in agreement with the hypothesis that evolutionary neocentromeres tend to be ‘stabilized’ through the recruitment of satellite DNA. Indeed, it has been proposed that the mosaicism observed for some clinical neocentromeres may be due to their intrinsic mitotic instability (Marshall et al. 2008). On the contrary, the neocentromere of horse chromosome 11 must be sufficiently stable to be present in all individuals of the species. Heterochromatin has been shown to limit spreading of protein domains in *S. pombe* (Partridge et al. 2000) and to specifically exclude CENP-A incorporation in *Drosophila* (Heun et al. 2006). In addition, although the role of the centromeric protein CENP-B is not well understood, it has been suggested that this protein might contribute to the organization of centromeric heterochromatin both in fission yeast (Nakagawa et al. 2002) and in humans (Okada et al. 2007). Since we did not find any evidence for the presence of CENP-B boxes (the consensus sequence binding CENP-B) in the ECA11 centromeric region (data not shown), it is tempting to speculate that the absence or low level of binding to chromatin of this protein may contribute to the sliding of CENP-A domains described here. We propose that fluctuations in CENP-A nucleosome positioning may give rise to a diffusion-like behaviour, a form of un-anchored chromatin spreading, that could account for ‘centromere sliding’. Such dynamic behaviour might be one reason for the great variability of centromere-associated DNA sequences.

It is worth noticing that cytogenetic approaches on metaphase chromosomes never revealed positional variation of the primary constriction on horse chromosome 11, indicating that the polymorphism described here involves a defined genomic region whose size is under the resolution limit of cytogenetic analysis; indeed, this region occupies about 500 kb. In any case, the phenomenon described here is distinct from larger scale centromere repositioning observed during karyotype evolution (Carbone et al. 2006; Rocchi et al. 2012).

It is possible that the centromere studied here is particularly dynamic because it is evolutionarily young and lacks satellite tandem repeats (Wade et al. 2009; Piras et al. 2010). As mentioned above, some positional variation, affecting centromeric domains on alphoid DNA, was observed on the mature human chromosome 17 (Maloney et al. 2012). In that case, two adjacent alpha satellite arrays were shown to possess centromere activity. In our system, the lack of satellite DNA at the centromere of horse chromosome 11 is a stable feature in all individuals of the horse species and was maintained for many generations during evolution; therefore, the mechanisms of satellite DNA recruitment and the precise role of repetitive sequences in centromere function and stabilization remain to be established. Satellite DNA recruitment appears to be a late step in centromere repositioning events, with repetitive DNA arrays proposed to play a role in stabilizing centromere position. We suggest that the colonization of a CENP-A domain by satellite DNA may progressively reduce the positional flexibility of the centromere through a satellite-mediated stabilization mechanism.

We do not know the probability of centromere movement per cell per generation nor how far from their original position CENP-A binding domains can move. We have evidence that the position of these domains is endowed with a certain degree of stability as we did not detect any positional variation in our fibroblast cell lines at different culture passages (data not shown). Another open question is the evolutionary timescale of centromere movement; the great variability of CENP-A domain position in our ten chromosome sample suggests that this phenomenon is quite frequent, at least in horse chromosome 11.

We previously described, in non-horse species of the genus *Equus*, a number of centromeres at different maturation stages, some of which seem to be devoid of extended clusters of tandemly repeated DNA (Piras et al. 2010). These satellite-less equid centromeres represent a new and powerful model system offering a clear advantage with respect to engineered or clinical neocentromeres: They are natural, stably present in all individuals of a given species and can therefore be used as an ideal tool to study the maturation and fixation of evolutionary new centromeres. In addition, the non-repetitive nature of a number of equid centromeres and the availability of the complete sequence of the horse genome provide the chance to analyse, at the molecular level, the architecture, plasticity and evolution of natural centromeres.

## Electronic supplementary material

Below is the link to the electronic supplementary material.

ESM 1 (DOCX 73 kb)

ESM 2 (GIF 67 kb)

(TIFF 651 kb)

ESM 3 (GIF 56 kb)

(TIFF 312 kb)

ESM 4 (GIF 173 kb)

(TIFF 1647 kb)

ESM 5 (GIF 191 kb)

(TIFF 1602 kb)

ESM 6 (DOC 53 kb)

ESM 7 (DOCX 26 kb)

ESM 8 (DOC 133 kb)
