# Supplementary material for: Centromere sliding on a mammalian chromosome
Source: Chromosoma. 2014 Nov 21;124(2):277–87. doi: 10.1007/s00412-014-0493-6 (PMC4446527; doi:10.1007/s00412-014-0493-6)
Supplement: Supplementary file 1 — (DOCX 73 kb) [file 412_2014_493_MOESM1_ESM.docx]

**Supplementary material**

**Journal: Chromosoma**

**Article Title: Centromere Sliding on a Mammalian Chromosome**

Stefania Purgato^1^, Elisa Belloni^2^, Francesca M. Piras^2^, Monica Zoli^1^, Claudia Badiale^2^, Federico Cerutti^2^, Alice Mazzagatti^2^, Giovanni Perini^1^, Giuliano Della Valle^1^, Solomon G. Nergadze^2^, Kevin F. Sullivan^3^, Elena Raimondi^2*^, Mariano Rocchi^4*^, Elena Giulotto^2*^

^1^ Dipartimento di Farmacia e Biotecnologie (FABIT), Università di Bologna, Bologna, Italy

^2^ Dipartimento di Biologia e Biotecnologie “Lazzaro Spallanzani”, Università di Pavia, Pavia, Italy

^3^ Centre for Chromosome Biology, School of Natural Sciences, National University of Ireland, Galway, Ireland

^4^ Dipartimento di Biologia, Università di Bari, Bari, Italy

* Correspondence should be addressed to Elena Giulotto ([elena.giulotto@unipv.it](mailto:elena.giulotto@unipv.it)), Mariano Rocchi ([mariano.rocchi@uniba.it](mailto:mariano.rocchi@uniba.it)) or Elena Raimondi (elena.raimondi@unipv.it)

**Legends to Supplementary Figures**

**Figure S1. Chromosome analysis of the fibroblast cell lines.**

Metaphase spreads from the fibroblast cell lines of the five horses are shown. Chromosomes were stained by DAPI. Reverse images are shown.

**Figure S2. FISH localization of BACs**

Metaphase spreads were prepared from normal diploid horse fibroblasts. Chromosomes were stained with DAPI. BACs were labelled with CY3 (red) or Alexa488 (green). All the BACs are localized at the centromere of chromosome 11.

**Figure S3. BAC clones used in immuno-FISH experiments**

**a)** For each BAC clone used in immunoFISH experiments the coordinates on the horse reference genome sequence are reported. Different colours are used to distinguish the different horse cell lines.

**b)** The position of the BACs on the relevant region of horse chromosome 11 is indicated as a bar, using the same colour code as in panel **a)**.

**Figure S4. Dual colour immunolabelling of horse chromatin fibres**

Chromatin fibres were prepared from horse fibroblasts. Immunoreactions were carried out with an anti CENP-A monoclonal antibody (Abcam cat n. 13939), detected with a rhodamine conjugated secondary antibody (red) and with a CREST serum, detected with a fluoresceine conjugated secondary antibody (green). Fibres were stained with DAPI (blue).

**Legends to Supplementary Tables**

**Table S1. Primers used for q-PCR validation of ECA11 cen DNA enrichment**

**Table S2. Polymorphism analysis of HSF-D, HSF-G, HSF-E, HSF-C and HSF-B**

For each cell line (column 1) the table lists the position (column 2) of heterozygous SNPs identified by sequencing the PCR-products from total genomic DNA using the forward and reverse primers reported in columns 5 and 6. Column 3 reports the nucleotide call at the SNP position in input DNA. Column 4 reports the nucleotide call in CENP-A immunoprecipitated DNA. SNPs highlighted in blue are those reported in Figure 1 as blue carats and in Figure 3 as stars.

For HSF-B the position and primers are referred to a CA microsatellite locus.

**Table S3. Sequence analysis of the ECA11 centromeric region and of 64 control regions**
