# Supplementary figures and images for: Centromere sliding on a mammalian chromosome
Source: Chromosoma. 2014 Nov 21;124(2):277–87. doi: 10.1007/s00412-014-0493-6 (PMC4446527; doi:10.1007/s00412-014-0493-6)

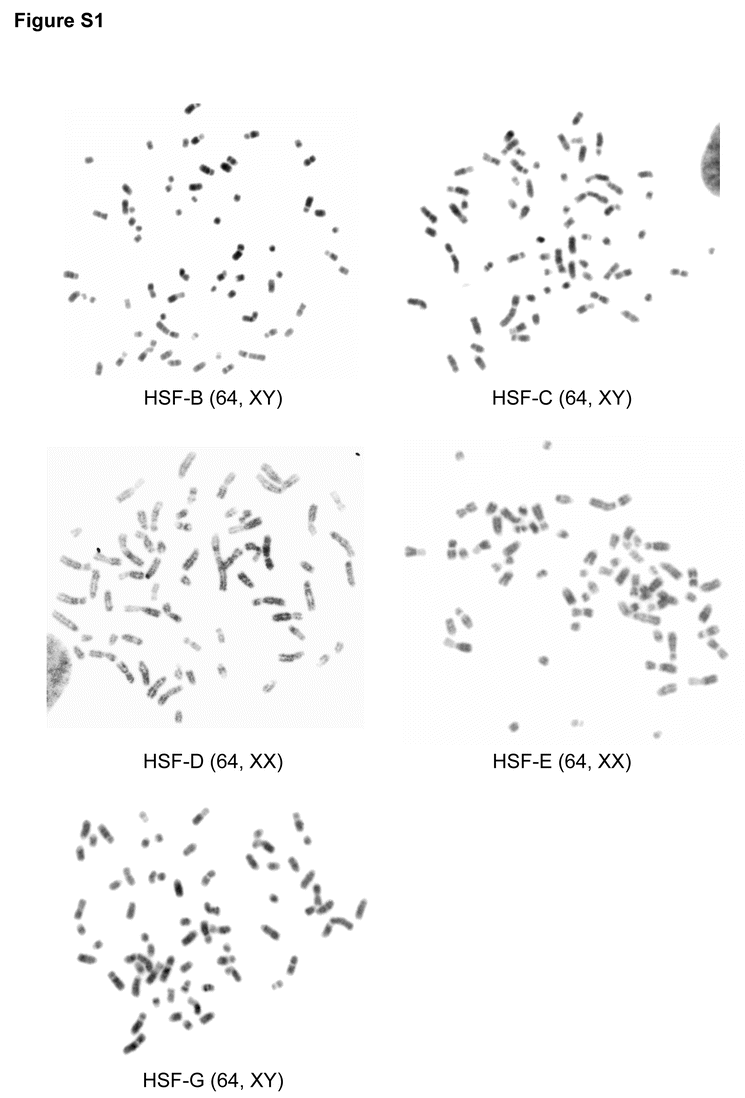

Supplement: Supplementary file 2 — (GIF 67 kb) [file 412_2014_493_Fig6_ESM.gif]

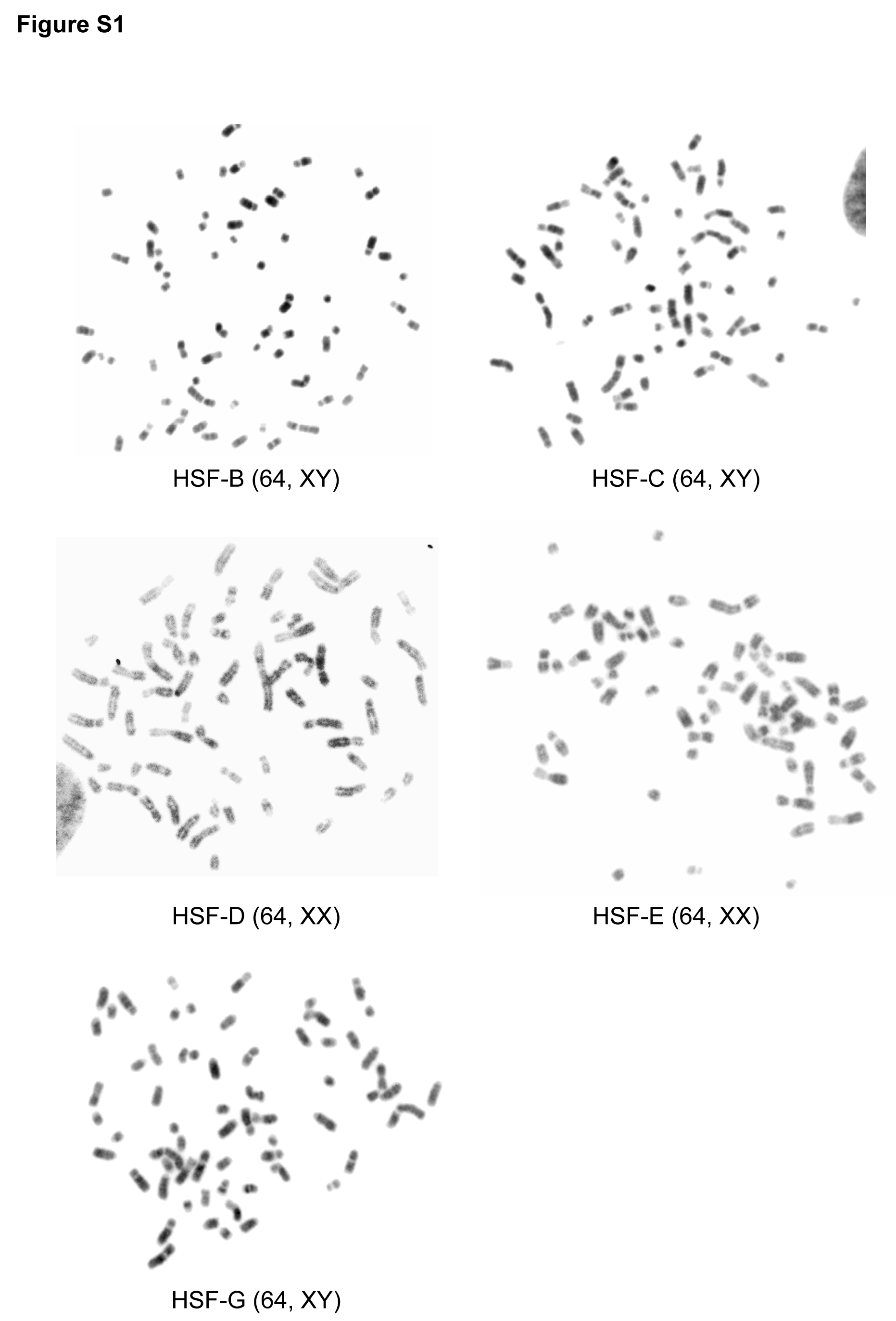

Supplement: Supplementary file 3 — (TIFF 651 kb) [file 412_2014_493_MOESM2_ESM.tif]

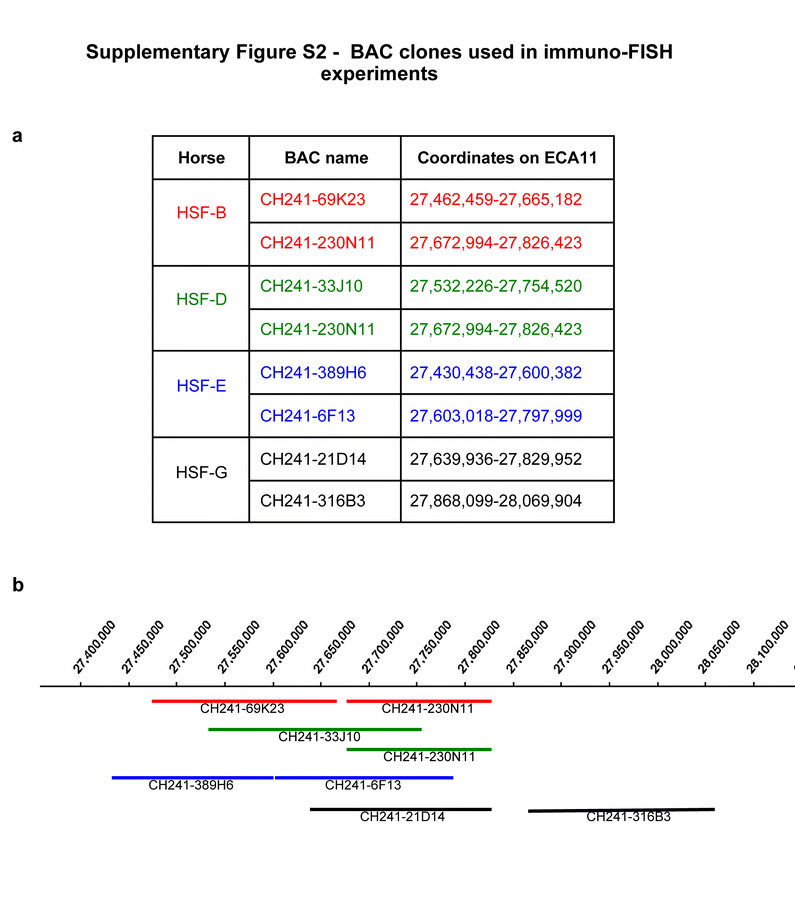

Supplement: Supplementary file 4 — (GIF 56 kb) [file 412_2014_493_Fig7_ESM.gif]

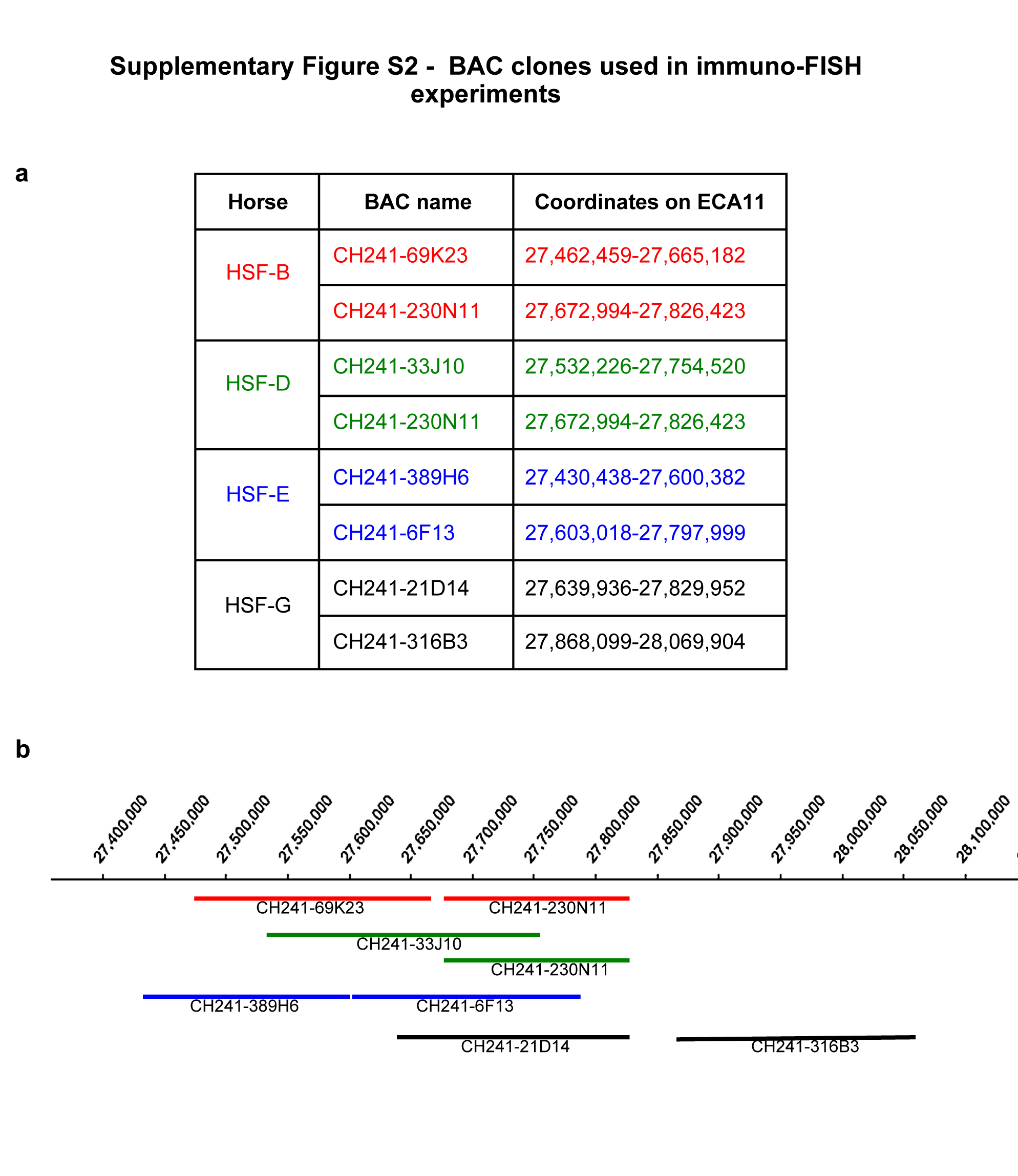

Supplement: Supplementary file 5 — (TIFF 312 kb) [file 412_2014_493_MOESM3_ESM.tif]

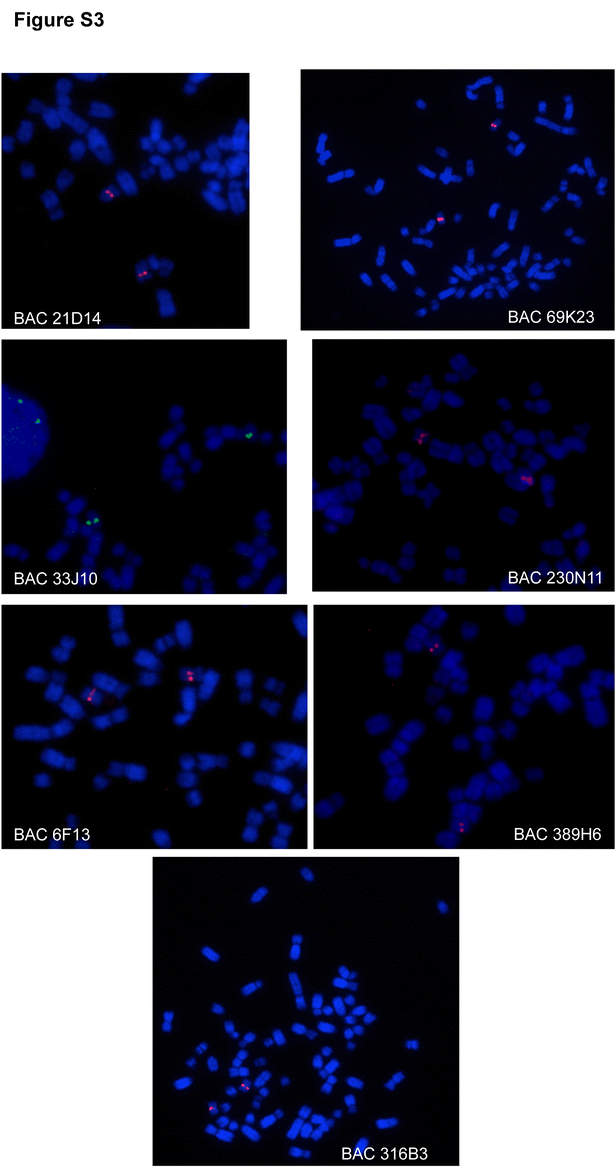

Supplement: Supplementary file 6 — (GIF 173 kb) [file 412_2014_493_Fig8_ESM.gif]

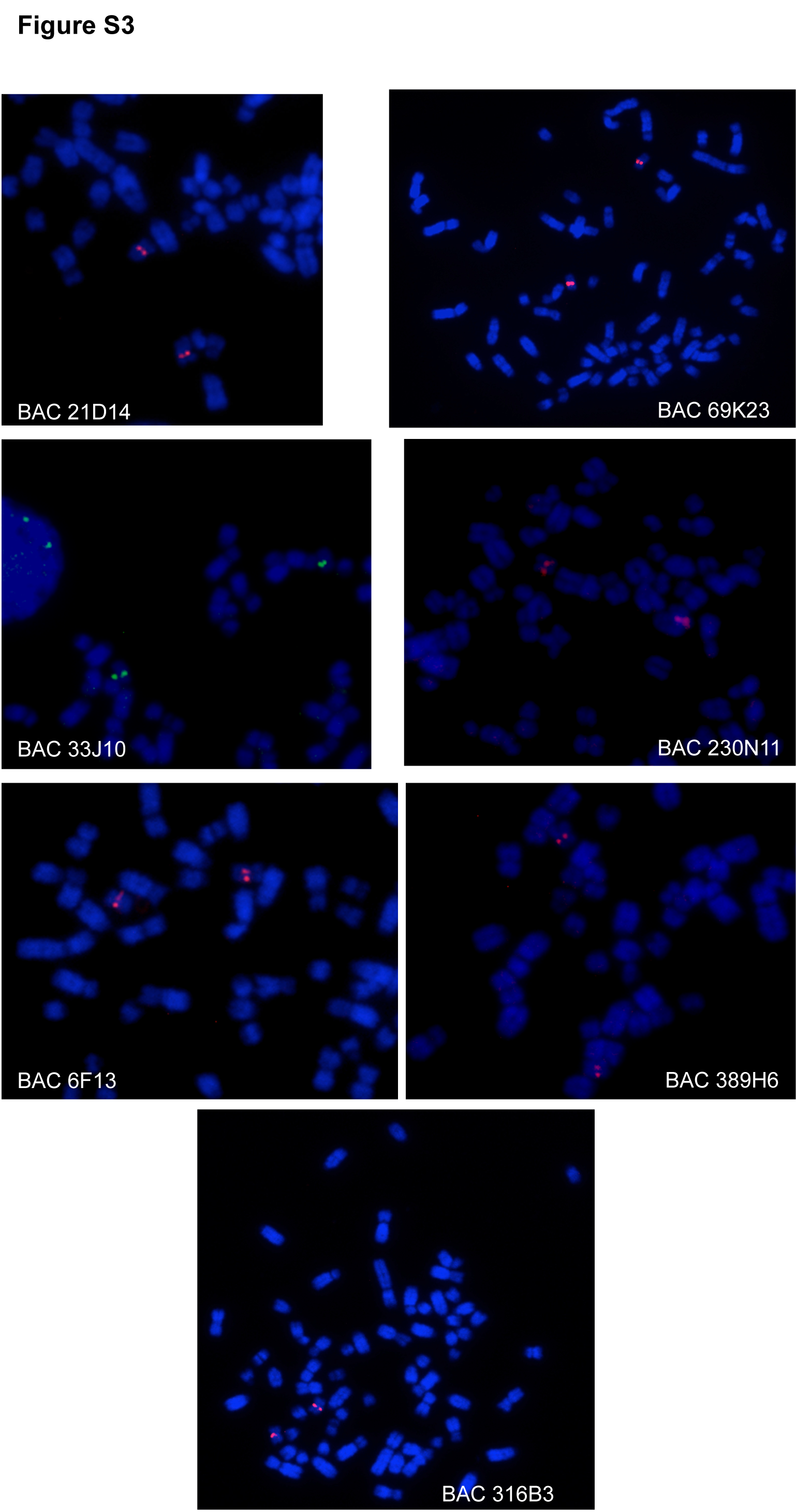

Supplement: Supplementary file 7 — (TIFF 1647 kb) [file 412_2014_493_MOESM4_ESM.tif]

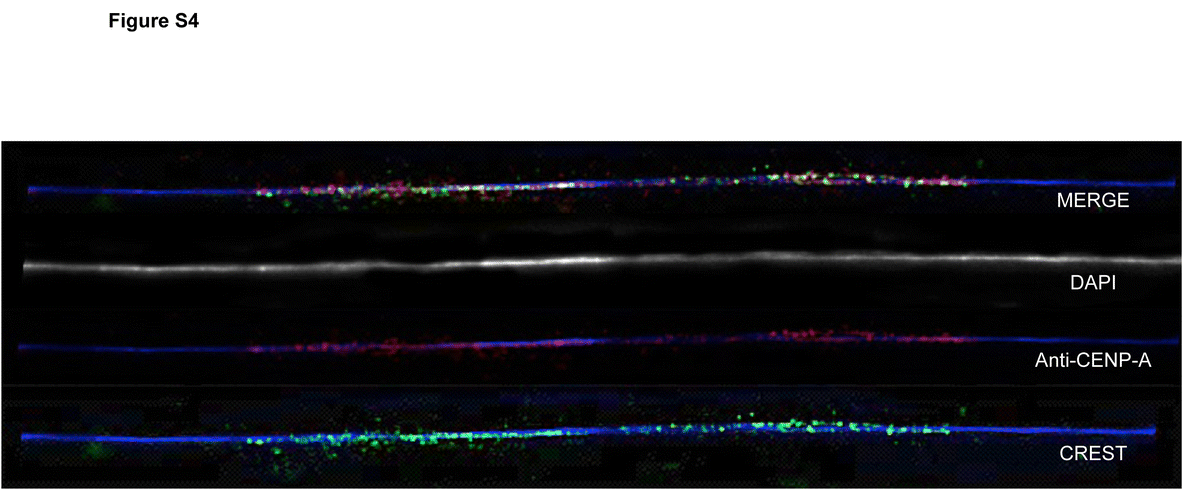

Supplement: Supplementary file 8 — (GIF 191 kb) [file 412_2014_493_Fig9_ESM.gif]

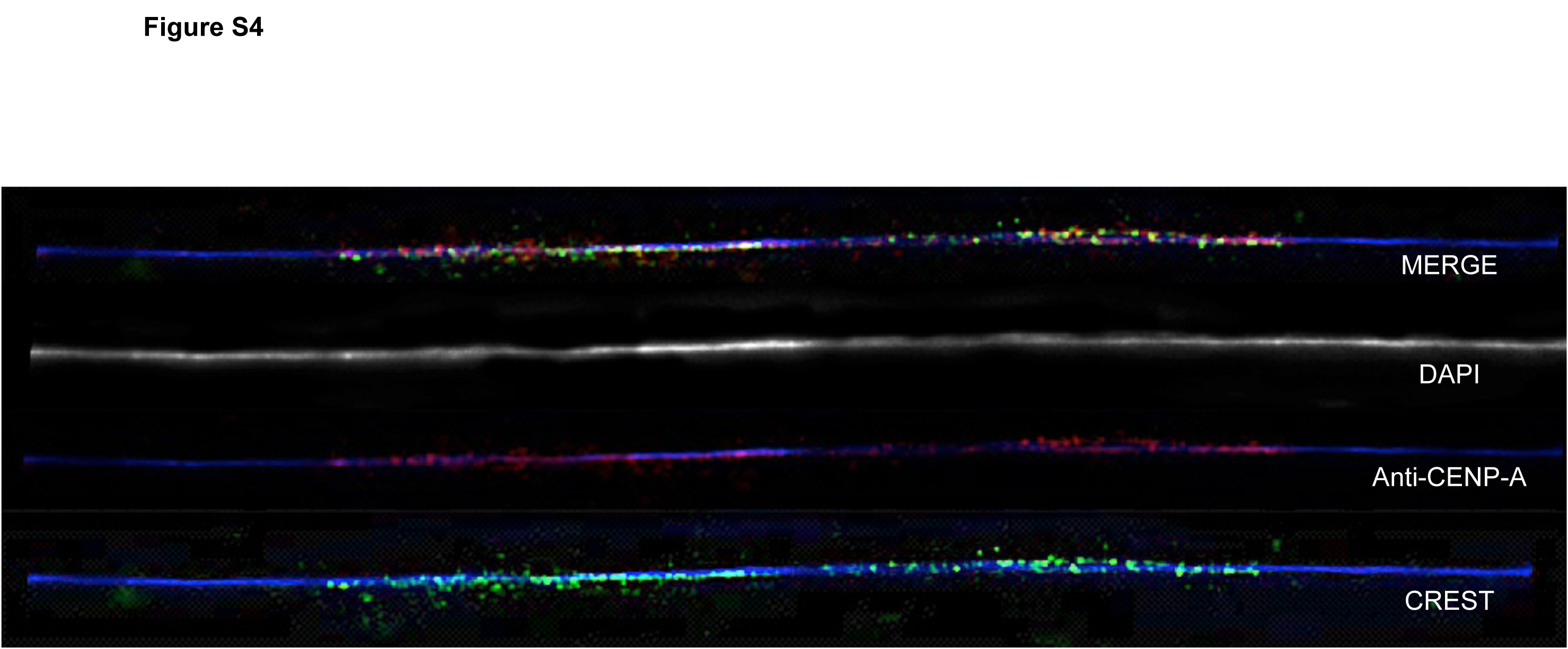

Supplement: Supplementary file 9 — (TIFF 1602 kb) [file 412_2014_493_MOESM5_ESM.tif]
