# Supplementary material for: Centromere sliding on a mammalian chromosome
Source: Chromosoma. 2014 Nov 21;124(2):277–87. doi: 10.1007/s00412-014-0493-6 (PMC4446527; doi:10.1007/s00412-014-0493-6)
Supplement: Supplementary file 10 — (DOC 53 kb) [file 412_2014_493_MOESM6_ESM.doc]

| **Number** | **Coordinates on ECA11** | **Forward primer** | **Reverse primer** |
| --- | --- | --- | --- |
| 1 | 27,489,994-27,490,094 | ATTGGGGGAGGAAACGTGA | GTGTCCGAAGTGGTAGTGGAG |
| 2 | 27,527,521-27,527,645 | ACACTCAAAGATCCCCATGC | GAATGGCTTTTCCGCTTTCC |
| 3 | 27,569,560-27,569,649 | ATGCCCTGGACTGTAAAACG | ATCCTCAAAGCTGAGCCAAA |
| 4 | 27,580,021-27,580,144 | GTCCTCATTTGCACGCTAGTTC | ATCAAGGGCTCCAAGTGACAGT |
| 5 | 27,599,541-27,599,650 | CCTCTTCTCATTTCATTCACTGC | AGAAATCAGATTGCGATGGGT |
| 6 | 27,623,127-27,623,247 | CTTCCCTTCCCTCTTCCTTCC | TGTCAACTTTTCATCTCCTTCCCT |
| 7 | 27,650,040-27,650,174 | AGCCACAATCAGTCCCAATG | CCCTCTTTTAGATGAGCACTGTC |
| 8 | 27,687,470-27,687,569 | GGAGGGCAAAGGTTACTTGGT | TCGAGAGAAATGCTGAAGTGCT |
| 9 | 27,687,704-27,687,797 | CAAAGCCTGGGAAAACACTC | CACGTGCCCCTGTTTTACTT |
| 10 | 27,720,646-27,720,775 | CAGCAAGGCATTTCCAGTGA | CATGCAAGACAAGGAGGAACG |
| 11 | 27,740,338-27,740,434 | CTACTTCTCCTCCAAAGCGTGT | ATGCACCTTCTCTGGATTCG |
| 12 | 27,760,909-27,761,022 | TCAGGATAGGGCACCGACTC | GCTCTTCAACATCTCCCTGCAA |
| 13 | 27,810,288-27,810,415 | GTCAGCCTGTACCGTGCAA | GCTGTGATTCAAAATGTGCCA |
| 14 | 27,821,187-27,821,291 | TTATGCAGGTGCAGCAAGTC | GCTTTGCCTTACCTCACTCG |
| 15 | 27,864,760-27,864,869 | TCTTCCCCTGTTGGTTTCAG | CTAGAAGGCTGGCAGATTGG |
| 16 | 27,877,774-27,877,885 | GGAAGTCGGGAGTTGTTGGTA | CCATGAAAAGCCTTGGTCCT |
| 17 | 27,910,140-27,910,272 | GAACATCTGCATTGGACTTGG | TGCATGAAGTTTCAAAGGAAGAG |
| 18 | 27,916,323-27,916,428 | CAGGGCCTGATGGAGAAATA | CATCATGGAAGAGGCGAAAT |
| 19 | 27,921,252-27,921,364 | AGGAAGAGCTGATTTGAGAGGAG | TGGATCAAATGCTACTTCCCTGT |
| 20 | 27,934,666-27,934,768 | GCTTCTCGCCCATATGAAAG | ATGTCCCAACGCTGAAAAAC |
| 21 | 27,935,028-27,935,132 | CCGACATCTTCAGTGATCCAAC | GTGCTCACTGGCTTCTCTCC |
| 22 | 27,966,050-27,966,138 | CATAACCCCTGGCATCCTTA | TGCCCCAGGGATAAATCATA |
| 23 | 27,966,325-27,966,444 | AAGGATTTCAAGGTGCTCAGGT | GGAATCCATAACTGTGCTGCAA |
| 24 | 27,985,955-27,986,054 | TCCACTTTCGACAACACTGC | ACGGACATACCGTTGCCTAC |
| 25 | 27,985,954-27,986,051 | CTCCACTTTCGACAACACTGC | GACATACCGTTGCCTACCACA |
| 26 | 27,990,583-27,990,679 | CTTTGCGCATGTCTCTCAAA | GCTGCACACAAAACGAAAGA |
| 27 | 28,008,830-28,008,953 | AGCCCTCCTTGAACTGTGGT | CCTCCTAAGAAGCCTGATCCTAC |

**Table S1: Primers used for q-PCR validation of ECA11 cen DNA enrichment**
