# Supplementary material for: Centromere sliding on a mammalian chromosome
Source: Chromosoma. 2014 Nov 21;124(2):277–87. doi: 10.1007/s00412-014-0493-6 (PMC4446527; doi:10.1007/s00412-014-0493-6)
Supplement: Supplementary file 11 — (DOCX 26 kb) [file 412_2014_493_MOESM7_ESM.docx]

**Table S2: Analysis of polymorphic alleles in HSF-D, -G , -E, -C and -B**

| **Horse** | **SNP position**  **on ECA11** | **Input** | **CENP-A** | **Forward primer** | **Reverse primer** |
| --- | --- | --- | --- | --- | --- |
| HSF-D | 27,643,838 | T/C | C | GTGTTTCAAGAGGAAGCAGT | GAGTGCTCACACTGACTCTG |
|  | 27,643,849 | C/T | C | GTGTTTCAAGAGGAAGCAGT | GAGTGCTCACACTGACTCTG |
|  | 27,691,524 | G/C | G/C | CTCAAGAGAGTTATGCTGGATG | TGCTAATCTGTTCTTCATCTCC |
|  | 27,691,648 | A/G | A/G | CATGAGGGATCTGTGTGATA | ATCACAACCAGGAAATTGAC |
|  | 27,691,649 | T/C | T/C | CATGAGGGATCTGTGTGATA | ATCACAACCAGGAAATTGAC |
|  | 27,728,443 | G/A | A | CAAGGGGAACTAGAGAAAAT | GTTCTTTAAAGGCTCAGAGGT |
|  | 27,728,445 | T/C | C | CAAGGGGAACTAGAGAAAAT | GTTCTTTAAAGGCTCAGAGGT |
|  | 27,744,615 | T/C | C | GACTAGGAGGGAGGAAGAAA | ACAATGAGTGTTGTGAATGC |
|  | 27,744,727 | T/G | G | TCACATTCTTTGTAGCTTGC | GGTGCATGACTTTTGAGATT |
| HSF-G | 27,728,254 | G/C | C | CAGAAAAGTATCCGTCAAGG | CCCACTTCAGTCTCATTACC |
|  | 27,728,473 | A/T | A | AGTGATGGTTACCAGAGGAA | GAGTTGGGAGCTGTTCTTTA |
|  | 27,743,389 | A/T | A | CCTATGTCTTGGAAGCACTC | CTATCCCCGGGTTAAATAAT |
|  | 27,937,408 | C/T | T | TTAAAGTGGCCAATCCTCCA | CCCTCGAGATCACAGGAAGT |
|  | 27,937,423 | T/C | C | TTAAAGTGGCCAATCCTCCA | CCCTCGAGATCACAGGAAGT |
|  | 27,937,428 | C/A | A | TTAGATCCCCAAATAACTGC | TCAGGTGATGATTGTTGCTA |
|  | 27,937,445 | A/G | G | TTAGATCCCCAAATAACTGC | TCAGGTGATGATTGTTGCTA |
| HSF-E | 27,545,464 | G/C | G | ATGTTTCATCCCCACACT | GCATTTGAGGGTTAATACTG |
|  | 25,545,512 | A/T | A | ATGTTTCATCCCCACACT | GCATTTGAGGGTTAATACTG |
|  | 27,586,213 | A/G | A | CTGAATTCTAAGCCCAAATC | GTGGCATCGGATGTTAGT |
|  | 27,586,264 | T/A | T | CTGAATTCTAAGCCCAAATC | GTGGCATCGGATGTTAGT |
|  | 27,604,956 | T/C | T | GACCTGCGGTTTCAGTAG | GGGACTTTTACCAAAAACTG |
|  | 27,604,966 | A/C | C | GACCTGCGGTTTCAGTAG | GGGACTTTTACCAAAAACTG |
|  | 27,604,968 | A/C | C | GACCTGCGGTTTCAGTAG | GGGACTTTTACCAAAAACTG |
|  | 27,623,701 | A/G | A/G | ATGAATCCTCTCTTTCCTG | TTACCCAACATTCCACAG |
|  | 27,623,708 | C/T | C/T | ATGAATCCTCTCTTTCCTG | TTACCCAACATTCCACAG |
|  | 27,623,738 | A/G | A/G | ATGAATCCTCTCTTTCCTG | TTACCCAACATTCCACAG |
|  | 27,623,756 | C/T | C/T | ATGAATCCTCTCTTTCCTG | TTACCCAACATTCCACAG |
|  | 27,623,763 | C/T | C/T | ATGAATCCTCTCTTTCCTG | TTACCCAACATTCCACAG |
|  | 27,643,849 | C/T | C/T | GTGTTTCAAGAGGAAGCAGT | GAGTGCTCACACTGACTCTG |
|  | 27,643,867 | G/A | G/A | GTGTTTCAAGAGGAAGCAGT | GAGTGCTCACACTGACTCTG |
|  | 27,648,737 | G/A | G/A | AGCAGCTATTATGTGGTGGT | GCAAAATCCTGTGCAATC |
|  | 27,648,753 | T/C | T/C | AGCAGCTATTATGTGGTGGT | GCAAAATCCTGTGCAATC |
|  | 27,691,648 | A/G | A | CATGAGGGATCTGTGTGATA | ATCACAACCAGGAAATTGAC |
|  | 27,691,649 | T/C | T | CATGAGGGATCTGTGTGATA | ATCACAACCAGGAAATTGAC |
|  | 27,713,448 | C/T | T | AGTAACTGGCAAGGAACAA | AAGCAGAAGAGCCAAGAG |
|  | 27,713,449 | G/C | C | AGTAACTGGCAAGGAACAA | AAGCAGAAGAGCCAAGAG |
| HSF-C | 27,598,926 | T/C | T | AGCGGTCACGTAGACAGACTC | CTTGTCCTCTATGTGAGCTCTTTCC |
|  | 27,599,158 | A/C | A | AGCGGTCACGTAGACAGACTC | CTTGTCCTCTATGTGAGCTCTTTCC |
|  | 27,599,338 | C/G | G | AGCGGTCACGTAGACAGACTC | CTTGTCCTCTATGTGAGCTCTTTCC |
|  | 27,599,532 | C/T | C | AGCGGTCACGTAGACAGACTC | CTTGTCCTCTATGTGAGCTCTTTCC |
|  | 27,680,582 | G/A | G | CTTTCTCTCTCTCACTCCCTCTGTC | TGGGATGAGGCATGTGTACC |
|  | 27,680,597 | G/A | G | CTTTCTCTCTCTCACTCCCTCTGTC | TGGGATGAGGCATGTGTACC |
|  | 27,899,278 | G/A | G | CAATGTATTCTGGTTTGCCTCTTT | CACTTTCTCCCTTTGAATCACTG |
| HSF-B | *27,572,885 | (CA)_13_/  (CA)_14_ | (CA)_14_ | GTTCCTTCTTTCCTCATCCTCC | TCCCTTCTCCCAGTTTCACC |

*Start site of the polymorphic microsatellite locus
