# Supplementary material for: Centromere sliding on a mammalian chromosome
Source: Chromosoma. 2014 Nov 21;124(2):277–87. doi: 10.1007/s00412-014-0493-6 (PMC4446527; doi:10.1007/s00412-014-0493-6)
Supplement: Supplementary file 12 — (DOC 133 kb) [file 412_2014_493_MOESM8_ESM.doc]

**Table S3: Sequence analysis of the ECA11 centromeric region and of 64 control regions**

| **Region** | **GC (%)** | **SINEs (%)** | **LINEs (%)** | **LTRs (%)** | **DNA Elements**  **(%)** | **Total Repeat**  **Count (%)** | **Small RNAs**  **(%)** | **Low Complexity**  **(%)** |
| --- | --- | --- | --- | --- | --- | --- | --- | --- |
| HSF-B Peak1 | 40.82 | 9.36 | 19.38 | 8.68 | 3.45 | 41.16 | 3.87 | 0.13 |
| HSF-B peak2 | 39.68 | 8.86 | 17.65 | 13.21 | 6.18 | 45.90 | 3.65 | 0.16 |
| HSF-C Peak1 | 39.79 | 9.31 | 21.22 | 8.45 | 4.05 | 43.27 | 3.94 | 0.14 |
| HSF-C Peak2 | 38.14 | 8.86 | 21.36 | 9.22 | 3.62 | 43.07 | 4.72 | 0.18 |
| HSF-D | 39.76 | 8.92 | 22.94 | 10.17 | 4.39 | 46.51 | 4.05 | 0.11 |
| HSF-E | 40.21 | 8.82 | 19.99 | 8.17 | 3.78 | 41.00 | 3.72 | 0.12 |
| HSF-G Peak1 | 39.35 | 7.67 | 20.91 | 12.57 | 5.58 | 46.73 | 3.59 | 0.16 |
| HSF-G Peak2 | 38.32 | 10.63 | 13.55 | 8.33 | 7.57 | 40.32 | 5.45 | 0.12 |
| Whole region  chr11:27,514,628-  28,049,577 nt | 39.12 | 8.66 | 19.78 | 10.99 | 4.87 | 44.41 | 3.90 | 0.14 |
|  | | | | | | | | |
| Upstream Ctrl  chr11:26,679,679-  27,214,628 nt | 43.34 | 9.67 | 18.56 | 8.23 | 4.86 | 41.41 | 3.84 | 0.11 |
| Downstream Ctrl  chr11:28,249,577-28,784,526 nt | 36.63 | 8.00 | 24.65 | 9.43 | 3.80 | 45.88 | 4.56 | 0.19 |
| Ctrl region 1  chr1:16,249,577-16,784,526 nt | 34.74 | 5.44 | 36.80 | 7.55 | 3.44 | 53.24 | 3.08 | 0.10 |
| Ctrl region 2  chr1:100,249,577-100,784,526 nt | 39.52 | 7.25 | 16.64 | 4.90 | 3.77 | 32.59 | 3.18 | 0.20 |
| Ctrl region 3  chr2:16,249,577-16,784,526 nt | 48.12 | 9.48 | 19.49 | 5.82 | 4.76 | 39.61 | 2.03 | 0.13 |
| Ctrl region 4  chr2:101,249,577-101,784,526 nt | 37.24 | 7.02 | 21.03 | 10.27 | 4.14 | 42.50 | 3.99 | 0.05 |
| Ctrl region 5  chr3: 16,249,577-16,784,526 nt | 44.92 | 9.24 | 19.09 | 7.19 | 3.88 | 39.54 | 2.29 | 0.34 |
| Ctrl region 6  chr3:101,249,577-101,784,526 nt | 38.20 | 7.37 | 22.51 | 9.80 | 5.15 | 45.19 | 1.92 | 0.26 |
| Ctrl region 7  chr4: 14,249,577-14,784,526 nt | 41.49 | 5.79 | 32.34 | 4.42 | 3.64 | 46.20 | 3.84 | 0.07 |
| Ctrl region 8  chr4: 84,249,577-84,784,526 nt | 42.74 | 10.46 | 19.43 | 3.56 | 5.50 | 38.95 | 7.11 | 0.21 |
| Ctrl region 9  chr5: 14,249,577-14,784,526 nt | 42.88 | 7.01 | 21.47 | 9.86 | 4.67 | 43.10 | 3.70 | 0.06 |
| Ctrl region 10  chr5: 84,249,577-84,784,526 nt | 38.97 | 7.45 | 25.02 | 7.92 | 3.89 | 44.41 | 4.03 | 0.15 |
| Ctrl region 11  chr6:16,249,577-16,784,526 nt | 38.08 | 6.91 | 21.88 | 11.35 | 3.74 | 43.93 | 3.63 | 0.14 |
| Ctrl region 12  chr6:66,249,577-66,784,526 nt | 41.88 | 6.98 | 22.13 | 6.52 | 2.44 | 38.08 | 4.20 | 0.22 |
| Ctrl region 13  chr7:16,249,577-16,784,526 nt | 37.15 | 7.25 | 30.14 | 7.02 | 4.01 | 48.45 | 3.86 | 0.20 |
| Ctrl region 14  chr7: 64,249,577-64,784,526 nt | 39.47 | 12.26 | 21.77 | 8.59 | 3.45 | 46.29 | 2.33 | 0.14 |
| Ctrl region 15  chr8:16,249,577-16,784,526 nt | 48.47 | 9.89 | 16.68 | 7.94 | 5.79 | 40.42 | 3.21 | 0.17 |
| Ctrl region 16  chr8: 64,249,577-64,784,526 nt | 42.91 | 7.08 | 15.01 | 6.17 | 5.44 | 33.89 | 1.52 | 0.13 |
| Ctrl region 17  chr9:16,249,577-16,784,526 nt | 37.90 | 6.74 | 20.39 | 8.06 | 5.16 | 40.45 | 3.73 | 0.07 |
| Ctrl region 18  chr9: 65,249,577-65,784,526 nt | 45.63 | 10.02 | 15.96 | 10.06 | 4.88 | 40.91 | 3.42 | 0.10 |
| Ctrl region 19  chr10:11,249,577-11,784,526 nt | 41.64 | 5.32 | 31.93 | 12.48 | 2.41 | 52.14 | 1.97 | 0.32 |
| Ctrl region 20  chr10: 65,249,577-65,784,526 nt | 37.64 | 6.48 | 21.21 | 6.34 | 4.66 | 38.69 | 3.35 | 0.16 |
| Ctrl region 21  chr12:7,249,577-7,784,526 nt | 37.97 | 8.90 | 19.74 | 12.96 | 3.10 | 44.69 | 2.65 | 0.21 |
| Ctrl region 22  chr12: 23,149,577-23,684,526 nt | 38.93 | 4.28 | 39.48 | 14.61 | 2.84 | 61.35 | 3.02 | 0.10 |
| Ctrl region 23  chr13:7,249,577-7,784,526 nt | 40.60 | 3.90 | 35.18 | 12.78 | 2.01 | 53.87 | 2.07 | 0.07 |
| Ctrl region 24  chr13: 23,149,577-23,684,526 nt | 45.21 | 10.32 | 17.99 | 10.63 | 6.92 | 45.97 | 3.03 | 0.16 |
| Ctrl region 25  chr14:25,349,577-25,884,526 nt | 38.95 | 9.93 | 27.51 | 8.34 | 4.58 | 50.50 | 2.76 | 0.05 |
| Ctrl region 26  chr14:66,249,577-66,784,526 nt | 35.92 | 5.68 | 18.25 | 7.40 | 4.32 | 35.79 | 2.98 | 0.25 |
| Ctrl region 27  chr15: 25,349,577-25,884,526 nt | 34.97 | 5.91 | 27.02 | 10.31 | 2.80 | 46.07 | 4.41 | 0.13 |
| Ctrl region 28  chr15:66,249,577-66,784,526 nt | 43.25 | 9.63 | 19.26 | 10.10 | 5.27 | 44.29 | 2.93 | 0.18 |
| Ctrl region 29  chr16: 25,349,577-25,884,526 nt | 40.50 | 10.78 | 18.88 | 6.62 | 5.68 | 42.15 | 3.84 | 0.13 |
| Ctrl region 30  chr16:66,249,577-66,784,526 nt | 39.93 | 6.95 | 24.77 | 7.00 | 4.88 | 43.60 | 3.18 | 0.07 |
| Ctrl region 31  chr17: 25,349,577-25,884,526 nt | 40.47 | 8.18 | 19.87 | 9.31 | 5.37 | 42.73 | 4.44 | 0.12 |
| Ctrl region 32  chr17:66,249,577-66,784,526 nt | 39.87 | 6.70 | 20.46 | 6.48 | 4.00 | 37.86 | 3.09 | 0.11 |
| Ctrl region 33  chr18: 25,349,577-25,884,526 nt | 35.23 | 5.91 | 23.86 | 9.35 | 4.24 | 43.40 | 3.65 | 0.08 |
| Ctrl region 34  chr18:66,249,577-66,784,526 nt | 36.80 | 7.31 | 30.44 | 6.86 | 4.22 | 48.97 | 4.39 | 0.06 |
| Ctrl region 35  chr19:16,249,577-16,784,526 nt | 38.75 | 7.45 | 17.93 | 10.44 | 5.43 | 41.42 | 3.76 | 0.15 |
| Ctrl region 36  chr19:46,249,577-46,784,526 nt | 37.62 | 6.69 | 23.78 | 7.08 | 4.80 | 42.56 | 3.39 | 0.20 |
| Ctrl region 37  chr20:16,249,577-16,784,526 nt | 39.11 | 7.60 | 20.37 | 4.33 | 4.67 | 36.97 | 4.35 | 0.14 |
| Ctrl region 38  chr20:46,249,577-46,784,526 nt | 37.21 | 6.41 | 21.86 | 8.27 | 4.56 | 41.27 | 2.76 | 0.09 |
| Ctrl region 39  chr21:16,249,577-16,784,526 nt | 38.61 | 8.19 | 24.95 | 7.77 | 6.19 | 47.29 | 5.31 | 0.24 |
| Ctrl region 40  chr21:46,249,577-46,784,526 nt | 38.71 | 5.99 | 19.55 | 12.45 | 3.26 | 41.29 | 3.46 | 0.08 |
| Ctrl region 41  chr22:16,249,577-16,784,526 nt | 39.28 | 6.13 | 16.32 | 7.40 | 3.92 | 34.02 | 2.60 | 0.24 |
| Ctrl region 42  chr22:36,249,577-36,784,526 nt | 51.22 | 6.81 | 16.22 | 11.97 | 5.71 | 40.71 | 2.64 | 0.14 |
| Ctrl region 43  chr23:14,249,577-14,784,526 nt | 38.11 | 7.47 | 26.96 | 6.15 | 4.01 | 44.84 | 4.65 | 0.20 |
| Ctrl region 44  chr23:36,249,577-36,784,526 nt | 39.16 | 7.71 | 12.22 | 4.29 | 5.90 | 30.23 | 4.29 | 0.17 |
| Ctrl region 45  chr24:14,249,577-14,784,526 nt | 42.22 | 7.99 | 16.38 | 3.42 | 4.21 | 32.12 | 2.73 | 0.05 |
| Ctrl region 46  chr24:34,249,577-34,784,526 nt | 44.70 | 7.28 | 21.72 | 6.54 | 5.12 | 40.68 | 3.42 | 0.11 |
| Ctrl region 47  chr25:14,249,577-14,784,526 nt | 43.35 | 9.48 | 17.00 | 5.46 | 4.73 | 36.68 | 4.27 | 0.16 |
| Ctrl region 48  chr25:26,249,577-26,784,526 nt | 37.57 | 6.50 | 33.80 | 7.31 | 2.04 | 49.65 | 3.07 | 0.10 |
| Ctrl region 49  chr26:16,249,577-16,784,526 nt | 38.54 | 5.68 | 18.81 | 12.71 | 4.42 | 41.66 | 3.41 | 0.10 |
| Ctrl region 50  chr26:26,249,577-26,784,526 nt | 39.33 | 7.71 | 14.16 | 9.71 | 4.96 | 36.60 | 3.58 | 0.05 |
| Ctrl region 51  chr27:14,249,577-14,784,526 nt | 42.24 | 6.56 | 22.19 | 10.76 | 2.66 | 42.25 | 2.99 | 0.12 |
| Ctrl region 52  chr27:26,249,577-26,784,526 nt | 41.93 | 8.04 | 22.71 | 9.57 | 5.82 | 46.20 | 5.63 | 0.08 |
| Ctrl region 53  chr28:16,249,577-16,784,526 nt | 36.22 | 6.75 | 22.57 | 8.48 | 4.70 | 42.57 | 3.46 | 0.16 |
| Ctrl region 54  chr28:26,249,577-26,784,526 nt | 40.87 | 8.51 | 19.92 | 8.47 | 4.91 | 41.88 | 2.06 | 0.21 |
| Ctrl region 55  chr29:10,249,577-10,784,526 nt | 39.35 | 6.03 | 24.96 | 5.09 | 3.47 | 39.55 | 3.45 | 0.20 |
| Ctrl region 56  chr29:20,249,577-20,784,526 nt | 43.01 | 6.36 | 14.19 | 10.28 | 3.16 | 34.20 | 2.72 | 0.13 |
| Ctrl region 57  chr30:10,249,577-10,784,526 nt | 39.36 | 7.33 | 22.81 | 7.32 | 4.52 | 41.99 | 4.04 | 0.08 |
| Ctrl region 58  chr30:20,249,577-20,784,526 nt | 36.45 | 9.08 | 18.00 | 11.21 | 4.27 | 42.66 | 6.88 | 0.24 |
| Ctrl region 59  chr31:8,249,577-8,784,526 nt | 48.52 | 5.49 | 9.53 | 4.84 | 2.67 | 22.53 | 3.82 | 0.17 |
| Ctrl region 60  chr31:18,249,577-18,784,526 nt | 40.10 | 7.51 | 15.30 | 5.69 | 3.83 | 32.33 | 3.55 | 0.17 |
| Ctrl region 61  chrX:16,249,577-16,784,526 nt | 40.57 | 7.41 | 18.04 | 7.73 | 7.71 | 40.97 | 4.06 | 0.06 |
| Ctrl region 62  chrX:100,249,577-100,784,526 nt | 35.74 | 5.53 | 28.00 | 13.74 | 3.18 | 50.54 | 2.73 | 0.18 |
| Ctrl region - Mean values | 40.25 | 7.45 | 21.83 | 8.35 | 4.35 | 40.07 | 3.50 | 0.14 |
